# Genetic Analysis of Dengue Virus in Severe and Non-Severe Cases in Dhaka, Bangladesh, in 2018–2022

**DOI:** 10.3390/v15051144

**Published:** 2023-05-10

**Authors:** Rummana Rahim, Abu Hasan, Juthamas Phadungsombat, Nazmul Hasan, Nikhat Ara, Suma Mita Biswas, Emi E. Nakayama, Mizanur Rahman, Tatsuo Shioda

**Affiliations:** 1 Evercare Hospital Dhaka (Ex Apollo Hospitals Dhaka), Plot-81, Block-E, Bashundhara R/A, Dhaka 1229, Bangladesh; rummana.rahim@evercarebd.com (R.R.); rasel.hasan@evercarebd.com (A.H.); nazmul1.hasan@evercarebd.com (N.H.); nikhat.ara@evercarebd.com (N.A.); suma.biswas@evercarebd.com (S.M.B.); 2 Research Institute for Microbial Diseases, Osaka University, Suita 565-0781, Japan; juthamas@biken.osaka-u.ac.jp (J.P.); emien@biken.osaka-u.ac.jp (E.E.N.)

**Keywords:** DENV, Bangladesh, serotypes, phylogenetic tree, clades

## Abstract

Dengue virus (DENV) infections have unpredictable clinical outcomes, ranging from asymptomatic or minor febrile illness to severe and fatal disease. The severity of dengue infection is at least partly related to the replacement of circulating DENV serotypes and/or genotypes. To describe clinical profiles of patients and the viral sequence diversity corresponding to non-severe and severe cases, we collected patient samples from 2018 to 2022 at Evercare Hospital Dhaka, Bangladesh. Serotyping of 495 cases and sequencing of 179 cases showed that the dominant serotype of DENV shifted from DENV2 in 2017 and 2018 to DENV3 in 2019. DENV3 persisted as the only representative serotype until 2022. Co-circulation of clades B and C of the DENV2 cosmopolitan genotype in 2017 was replaced by circulation of clade C alone in 2018 with all clones disappearing thereafter. DENV3 genotype I was first detected in 2017 and was the only genotype in circulation until 2022. We observed a high incidence of severe cases in 2019 when the DENV3 genotype I became the only virus in circulation. Phylogenetic analysis revealed clusters of severe cases in several different subclades of DENV3 genotype I. Thus, these serotype and genotype changes in DENV may explain the large dengue outbreaks and increased severity of the disease in 2019.

## 1. Introduction

Dengue is widely considered to be the most important arthropod-borne viral disease as more than 75% of the world’s population is at risk of dengue infection. Indeed, the increase in globalization and business and leisure travel has markedly increased the risk of infection [1,2]. Dengue is caused by four serotypes of dengue virus (DENV1, DENV2, DENV3, and DENV4) in the Flaviviridae family [3]. The genomic RNA of DENV encodes three structural proteins (C, prM, and E) and seven non-structural proteins (NS1, NS2A, NS2B, NS3, NS4A, NS4B, and NS5) [4]. At both the 5′ and 3′ ends, the DENV open reading frame is flanked by untranslated regions (5′-UTR and 3′-UTR). The DENV genome exhibits a high degree of diversity. 

Although antigenic heterogeneity may exist within each serotype, the causes, prevalence, and impact of this heterogeneity remain unclear. Sequence-based typing of DENV has revealed the existence of canonical clades termed ‘genotypes’ [5,6], and each serotype has four to six geographically distinct genotypes. DENV1 comprises genotypes I, II, III (sylvatic), IV, V, and VI [7,8]. DENV2 comprises Asian-I, Asian-II, Asian/American, American, cosmopolitan, and sylvatic genotypes [9,10,11]. DENV3 comprises genotypes I, II, III, IV, and V [12], and DENV4 comprises genotypes I, IIA, IIB, III, and sylvatic [13,14,15]. Specific genotypes have been associated with mild or severe disease, and heterogeneous neutralization titers suggest that the immune response to some genotypes is more cross-protective than it is to others [16,17]. It is generally considered that these intra-serotype differences are relatively unimportant compared to the inter-serotype differences, and that intra-serotype antigenic variation contributes to genotype-specific case outcomes and epidemic patterns [18].

In Bangladesh, DENV3 was isolated for the first time from a patient in 1964 [19] and was found to be the main serotype in circulation during the outbreaks between 2000 and 2002 [20]. Thereafter, and until 2012, serotype data were not recorded properly, and limited data are available for that period. During the period 2013–2016, DENV1 and DENV2 were the predominant serotypes in circulation in Bangladesh [21]. Although only a limited number of DENV sequences have been reported from Bangladesh, co-circulation of all four serotypes has been reported in India [22].

The severity of dengue infection is affected by whether it differs from the previous predominant DENV serotype and/or genotype in circulation [23,24]. Consequently, the introduction of new serotypes or genotypes can lead to outbreaks of severe dengue. We previously reported that the dominant serotype shifted from DENV2 in 2017 and 2018 to DENV3 from 2019 until August 2022, when the only prevalent serotype was DENV3 (100%) and there was no evidence of other serotypes [25]. In the present study, we conducted a phylogenetic analysis of the envelope protein region of DENVs to determine the viral genotypes and evolutionary relationships among past and present isolates [26]. 

## 2. Materials and Methods

### 2.1. Ethical Approval

This dengue serotyping study was approved by the Research and Ethical Practice Committee of Evercare Hospital Dhaka (previously Apollo Hospitals Dhaka, approval number: ERC 16/2018-3). De-identified specimens stored at −80 °C were used with a different code for the present study. This study was exempt from obtaining participants’ consent since only leftover specimens were used after anonymization.

### 2.2. Patients and Clinical Specimens

Patients with clinical suspicion of dengue who visited Evercare Hospital Dhaka (previously Apollo Hospitals Dhaka) from 2018 to 2022 were included in this study, irrespective of their age. For serotype determination, patients with dengue PCR Threshold Cycle (Ct) value > 34 cycles were excluded from this study. Samples with Ct values < 30 in the serotyping PCR were included for sequencing in the present study. In 2019 and 2021, samples were randomly selected for sequencing, since there were more than 40 samples with Ct values < 30 in these years. As a routine diagnostic test, 3 mL and 0.5 mL to 1 mL of whole blood were collected from adult and pediatric patients, respectively, in plain vacutainers (red top). Serum was separated by centrifugation and samples were stored at −80 °C until RNA was extracted.

### 2.3. Detection of Dengue NS1 Antigen 

The NS1 antigen of DENV was detected using SD BIOLINE Dengue NS1 Ag kits (SD Bioline, Seoul, Republic of Korea). Three drops (100 µL) of serum sample was added to the kit and results were obtained within 15–20 min after comparison with the positive control line in the device. The presence of two colored lines (one for control, C, and one for test, T) in the result window indicates that the specimen is positive for the dengue NS1 antigen, and the presence of only the control line (C) indicates that the sample is negative.

### 2.4. RNA Extraction and Real-Time Reverse Transcriptase PCR

Two separate extraction kits were used for viral RNA extraction. The samples from 2018 to 2019 were assayed using a QIAamp MinElute Virus Spin Kit (Qiagen, Hilden, Germany), and the samples from 2020 to 2022 were assayed with a MagMax Viral/Pathogen kit (Thermo Fisher Scientific, Waltham, MA, USA). Viral RNA was extracted from 200 µL of serum following the manufacturers’ instructions and stored at −80 °C.

For samples from 2018 to 2019, a one-step reverse transcriptase real-time PCR kit (Fast Track Diagnostics, Esch-sur-Alzette, Luxembourg) was used for the detection of DENV. A total of 15 µL of PCR master mix containing 12.5 µL of buffer, 1.5 µL of primer–probe mix, and 1 µL of enzyme was prepared for each sample, negative control, and positive control. Then, 10 µL of the extracted RNA, nuclease-free water (negative control), and synthetic DNA (positive control) was added to a 0.1 mL PCR tube. PCR amplification was performed using a Rotor Gene Q Thermal Cycler (Qiagen). The amplification protocol consisted of 50 °C for 15 min followed by 94 °C for 1 min, and then 40 cycles of 94 °C for 8 s, and finally 60 °C for 1 min. Signal was acquired at 60 °C and analysis was performed using a linear scale. Thresholds were set manually on each run. For the target, any exponential curve crossing this threshold was considered positive. Fluorescence detected in the green channel was used for the amplification of DENV and the red channel was used for the internal control.

For samples from 2020 to 2022, a commercial one-step reverse transcriptase real-time PCR kit (TRUPCR Dengue Detection Kit, Bhopal, India) was used for the detection of DENV. A total of 15 µL of PCR master mix containing 10 µL of multiplex master mix, 2.65 µL of dengue primer–probe mix, 2 µL of internal control primer probe mix, and 0.35 µL of enzyme mix was prepared for each sample, and the negative and positive controls. Then, 10 µL of the extracted RNA from the samples and the negative and positive controls was separately added to a 0.2 mL PCR strip tube and amplified using a QuantStudio 5 Dx platform (Thermo Fisher Scientific) according to the manufacturer’s instructions. The PCR protocol used was 50 °C for 20 min and 94 °C for 10 min, followed by 45 cycles of 94 °C for 15 s, 55 °C for 30 s, and 72 °C for 30 s. Signal was acquired at 55 °C and analysis was performed using a linear scale. Thresholds were set manually for each run. Fluorescence detected in the FAM channel was used for amplification of Dengue virus and ROX channel was used for amplification of the internal control.

### 2.5. Serotype Specific Real-Time Reverse Transcriptase PCR

For dengue serotype identification, we used a commercial Genesig one-step reverse transcriptase real-time PCR kit from Primerdesign, UK. Four Dengue serotype-specific primer and TaqMan probe mixes were provided in a single tube and detected through four different channels, as described in materials provided with the kit. Briefly, 5 µL of RNA was added to a 15 µL mixture containing 10 µL of Oasig master mix, 1 µL of dengue primer probe mix, and 4 µL of nuclease-free water in a 0.2 mL PCR tube. Reverse transcription was performed using a Rotor Gene Q and QuantStudio 5 Dx platform at 55 °C for 10 min and enzyme activation at 95 °C for 2 min, followed by 50 cycles of denaturation at 95 °C for 10 s and annealing and extension together at 60 °C for 60 s. Different dengue serotypes could then be detected using different channels, as follows: DENV1 using the FAM channel, DENV2 using the VIC channel, DENV3 using the ROX channel, and DENV4 using the Cy5 channel, according to the kit manufacturer’s instruction.

### 2.6. Sequence Determination of the DENV Envelope Region

A total of 2.5 μL of DENV-positive RNA solution was added to 22.5 μL of master mix for reverse transcription followed by PCR amplification of the entire DENV envelope region using a One-step RT-PCR Kit (Qiagen) with a specific primer set (Table S1) in 25 μL reactions. The master mix contained 5 μL of 5× buffer, 1 μL of dNTP, 1 μL of enzyme, 0.75 μL of forward primer (20 μM), 0.75 μL of reverse primer (20 μM), and 14 μL of diethylpyrocarbonate (DEPC)-treated water. The QuantStudio 5 Dx platform was used with a temperature profile of 50 °C for 30 min followed by enzyme activation at 95 °C for 15 min and then 35 cycles of denaturation at 94 °C for 30 s, annealing at 55 °C for 60 s, and extension at 72 °C for 2 min 30 s. Samples were held for 10 min at 72 °C.

One microliter of the amplified product was mixed with 49 μL of master mix for nested PCR using PrimeSTAR GXL polymerase (Takara, Japan) for amplification in 50 μL reactions. The master mix contained 10 μL of 5× buffer, 4 μL of dNTP, 1 μL of enzyme, 1 µL of forward primer (20 μM), 1 μL of reverse primer (20 μM), and 32 μL of DEPC-treated water. The QuantStudio 5 Dx platform was used with the temperature profile of 94 °C for 1 min 30 s followed by 35 cycles of denaturation at 98 °C for 10 s, annealing at 55 °C for 30 s, and extension at 68 °C for 2 min. Samples were held for 7 min at 68 °C.

The DNA concentration of the amplified product was determined using a Nanodrop spectrophotometer (Thermo Fisher Scientific) before PCR purification was performed using a QIAquick gel extraction kit (Qiagen).

The purified nested PCR product was used for cycle sequencing with BigDye terminator ver 3.1 (Applied Biosystems, Waltham, MA, USA) and analyzed using an ABI 3130XL sequence analyzer. The details of the PCR and sequences of primer pairs are shown in Table S1. A total of 10 μL of sequencing reaction (master mix and sample) was used, consisting of 2 μL of big dye reaction mix, 1 μL of sequencing buffer, 1.6 μL (1 pmol) of primer, and 5.4 μL of distilled water containing up to 4 μL of DNA. The PCR protocol was as follows: 96 °C for 1 min, 25 cycles at 96 °C for 10 s, 50 °C for 5 s, and 60 °C for 4 min. The sequencing reaction was conducted according to the manufacturer’s protocol.

### 2.7. Phylogenetic Analysis of Envelope Region

The nucleotide sequence dataset was prepared by aligning the newly obtained DENV sequences and related sequences obtained using the NCBI Basic Local Alignment Search Tool (BLAST) [27]. Known genotype sequences were retrieved from GenBank [28] using AliView.

A total of 71 sequences were used for the phylogenetic analysis of DENV1 (Table S2). We included sequences for 12 strains reported earlier from Bangladesh and 9 sequences from the present study. The DENV1 genotyping dataset (1485 bp in length) included the following distinct genotypes: I (*n* = 11), II (*n* = 1), III (*n* = 1), IV (*n* = 5), V (*n* = 52), and VI (*n* = 1).

For the DENV2 dataset, a total of 168 sequences were used for the phylogenetic analysis (Table S2). We included 30 sequences from the present study and 67 reported sequences from Bangladesh. The DENV2 genotyping dataset (1485 bp in length) included the following five distinct genotypes: Asian-I (*n* = 4), Asian-II (*n* = 2), cosmopolitan clade A (*n* = 4), cosmopolitan clade B (*n* = 41), cosmopolitan clade C (*n* = 113), American (*n* = 1), and Asian/American (*n* = 3). For the DENV3 dataset, a total of 275 sequences were used for the phylogenetic analysis (Table S2). We included 140 sequences from the present study and 82 previously published sequences from Bangladesh. The DENV3 genotyping dataset (1479 bp in length) included the following five distinct genotypes: I (*n* = 218), II (*n* = 44), III (*n* = 6), IV (*n* = 3), and V (*n* = 4).

Phylogenetic trees were inferred from the alignment using the maximum likelihood (ML) method generated using W-IQ-TREE [29]. The best fit model was selected using ModelFinder [30], and an ultrafast bootstrap [31] with 1000 replicates was calculated. The phylogenetic tree was visualized using FigTree v1.4.4.

### 2.8. Mean Values of the Number of Nucleotide Differences among DENV3 E Regions

The number of nucleotide differences between any two sequences of DENV3 envelope regions in each year was counted, and its mean value among all the combinations was calculated.

### 2.9. Statistical Analysis

The collected data were processed and analyzed using SPSS software version 29 (IBM, Armonk, NY, USA). Differences in continuous variables were statistically evaluated using the Kolmogorov–Smirnov test. A *p* value of <0.05 was considered statistically significant. Highly significant *p* values were rounded off as <0.001.

## 3. Results

### 3.1. Dengue Demographic Data

A total of 3759 patients were evaluated for DENV from 2018 to 2022 at Evercare Hospital Dhaka (previously Apollo Hospital Dhaka), Bangladesh, and 839 cases were identified as dengue-positive. Samples with a dengue PCR Ct value <34 were used for the serotype determination study. Out of a total of 674 positive dengue cases, 495 serotype-positive cases were identified. In 2018, DENV1, DENV2, and DENV3 were detected and DENV2 was the dominant serotype (40.9%). From 2019, DENV3 was the predominant serotype (91.9%), with a much smaller percentage of DENV1 detected (8.1%), whereas DENV2 had totally disappeared. According to the findings of this study, DENV3 was the only serotype in circulation from 2020 to 2022. We performed sequencing of the dengue virus envelope region for a total of 179 cases in five consecutive years (Table 1 and Table S3). 

### 3.2. Population Demography

Over the five-year study period, DENV was most prevalent in patients aged 1–10 years in 2018, 2019, and 2022; however, in 2021, DENV was most prevalent in patients aged 11–20 years. Due to the COVID-19 pandemic and lockdown of the city, only a single DENV3 case (a 47-year-old male patient) was seen in 2020. Among the serotype-positive cases, males and females accounted for 297 (60%) and 198 (40%) of cases, respectively, with an M:F ratio of 3:2 (Table S4). According to the National Institute of Health USA guidelines [32], we divided the study population into children and adolescents (age < 18 years), adults (age 18 to 65 years), and the elderly (age > 65 years) and observed the serotype distribution among these groups. In 2018, DENV2 was the most prevalent serotype, being detected predominantly in adults (25.2%). In the 2019 outbreak, 60.5% of cases of the DENV3 serotype were detected among children and adolescents, and 29.8% and 38.8% of infected individuals in 2021 and 2022, respectively, were also children and adolescents (Table 1).

### 3.3. Severity and Serotype

We compared the severity (classical/severe dengue) in hospitalized patients during the period 2018–2022. Among 495 serotype-positive cases, 188 (38%) were hospitalized. Classical dengue presents as a fever without any warning signs (severe abdominal pain, persistent vomiting, marked change in body temperature; from fever to hypothermia). Dengue hemorrhagic fever (DHF), dengue shock syndrome (DSS), and dengue with severe thrombocytopenia were all considered as severe dengue.

Among hospitalized patients, the highest percentage of severe cases, i.e., 50.7% (35/69), was found in 2019, followed by 35.3% (12/34), 33.3% (7/21), and 20.3% (13/64) in 2022, 2021, and 2018, respectively. In 2018, among the three serotypes, the highest percentage of severe cases was observed in DENV3 cases (6/22, 27.3%) followed by DENV2 (6/29, 20.7%) and DENV1 cases (1/13, 7.7%). In the 2019 outbreak, the predominant serotype was DENV3, and the highest percentage of severe cases was recorded (35/64, 54.7%). In addition, five non-severe cases of DENV1 were also found. We found a single classical case of an outpatient with DENV3 in 2020 (Table 1).

We compared disease severity among 179 sequenced serotype cases including 81 inpatients and 98 outpatients. A total of 59 samples were sequenced in 2018, with severe DENV2 and DENV3 accounting for 6.78% (4/59) and 1.69% (1/59), respectively. In 2019, the dominant serotype shifted to DENV3 with an increased number of severe cases: 16 (39.02%) out of 41 sequenced cases. The only circulating serotype in 2020 (sequencing was not performed due to low viral load), 2021, and 2022 was DENV3. In 2021 and 2022, both classical and severe cases were reported with a proportion of severe cases of 15% (6/40) and 10.26% (4/39), respectively (Table S5).

### 3.4. Clinical Features and Complications of Hospitalized Severe Dengue Cases

All severe dengue cases presented with fever. The most common symptoms were nausea (53.7%), vomiting (53.7%), and abdominal pain (55.2%). Body ache and headache were reported in 34.3% and 23.9% of cases, respectively, while anorexia (17.9%), maculopapular skin rash (13.4%), weakness (11.9%), cough (10.4%), and diarrhea (9%) presented less frequently (Table 2).

Among 67 severe dengue cases, the most frequent complications were pleural effusion (29.9%) and breathing difficulties (17.9%). Bleeding manifestation was reported in 14.9% of cases. Ascitic fluid exudation was documented in 13.4% of cases. Hepatomegaly and splenomegaly were registered in 6% and 3% of cases, respectively. Among patients with breathing difficulties, mechanical ventilation was required for 4.5% (3/67) of patients who suffered from multi-organ failure and sepsis; of these patients, two (3%) died (Table 2).

### 3.5. Laboratory Findings of Hospitalized Severe Dengue Cases

The laboratory findings obtained for the hospitalized severe dengue cases are shown in Table 3. The table shows the complete blood counts and liver function tests for all patients. The most common abnormality was elevated liver enzymes (alanine transaminase; ALT and aspartate aminotransferase; AST), which was detected in 86.6% (58/67) of cases, along with hypoalbuminemia, which was found in 83.6% (56/67) of cases. A total of 59.7% (40/67) cases presented with thrombocytopenia (platelet count less than 50,000/cumm).

### 3.6. Phylogenetic Analysis of DENV1

**Figure 1:**
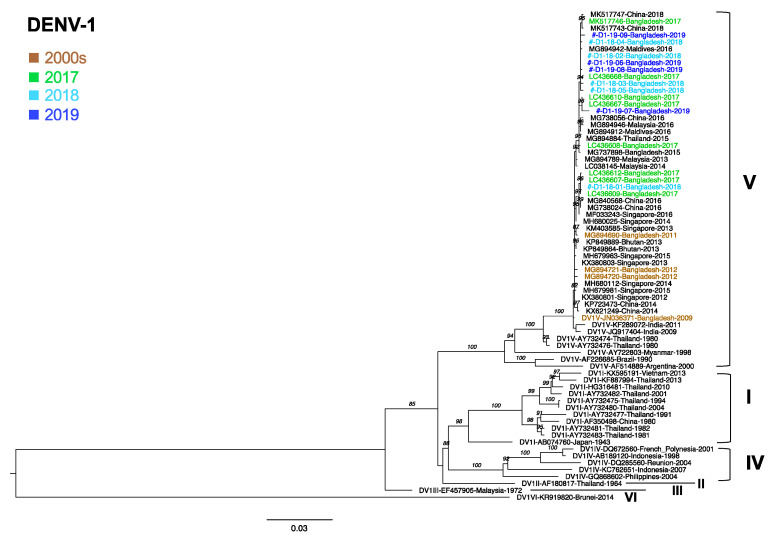
Genotyping of the DENV1 envelope region. A maximum likelihood phylogenetic tree was constructed using the W-IQ-TREE program in ModelFinder with ultrafast bootstrapping (UFBoot) and 1000 replicates. TIM2 +F+ G4 was selected as the best-fit model according to Bayesian information criteria. Data include DENV envelope-encoding sequences obtained in the present study (light blue for 2018 and royal blue for 2019) along with the sequences of known genotypes obtained from GenBank (green for 2017). The DENV sequences from Bangladesh for 2000 are brown. Viral genotypes are indicated to the right of sample names, which comprise the accession number, country, and reported year of each sequence. Numbers to the right of the branches are UFBoot support values exceeding 75%. Sequences of the present study were marked as #.

For the 40 DENV1-positive samples, we amplified the envelope region of 9 samples (5 from 2018 and 4 from 2019). All nine samples were classical dengue samples. Sequences from the present study were compared with previously circulating sequences [33]. We found that all of the Bangladesh DENV1 sequences were of genotype V (DENV1-V) and shared a common ancestor with the Thailand 1980 strain. DENV1 genotype V in Bangladesh had been circulating since 2009. All of the DENV1 sequences in the present study shared the same cluster with viruses from China, Singapore, Malaysia, India, and the Maldives. The strains most closely related to our DENV1-V strain were strains that circulated in Bangladesh in 2017 (LC436607 and others), the Maldives in 2016 (MG894942), and China in 2018 (MK517743 and MG517747) (Figure 1).

### 3.7. Phylogenetic Analysis of DENV2

**Figure 2:**
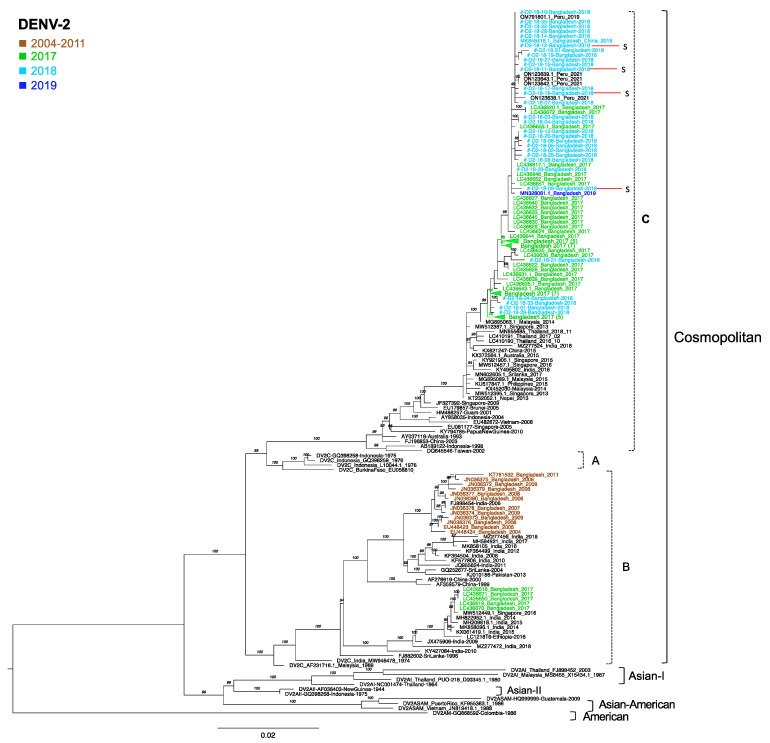
Genotyping of DENV2 in the envelope region. A maximum likelihood phylogenetic tree was constructed using the W-IQ-TREE program in ModelFinder with ultrafast bootstrapping (UFBoot) and 1000 replicates. TN+ F+ G4 was selected as the best-fit model according to Bayesian information criteria. Data include DENV envelope-encoding sequences obtained in the present study (light blue for 2018) and sequences for known genotypes obtained from GenBank (green for 2017 and royal blue for 2019). The DENV sequences from Bangladesh from 2004 to 2011 are brown. Viral genotypes are indicated to the right. Viral clade (A, B, and C) is indicated by dotted brackets. Sample names comprise the accession number, country, and reported year of each sequence. Numbers to the right of the branches are UFBoot support values exceeding 75%. Sequences of the present study were marked as #. Red lines indicate the severe cases.

For the DENV2 samples, we determined the nucleotide sequences of the envelope regions in 30 specimens that were sequenced successfully from 52 DENV2 specimens collected in 2018. Among the 30 DENV2 samples, 4 cases were severe (D2-18-09, D2-18-11, D2-18-12, and D2-18-16). All sequences obtained in the present study were genotyped as cosmopolitan and clustered in clade C [34,35]. These sequences formed a major cluster together with the Bangladesh sequences collected in 2017 and 2019. Furthermore, the viruses from Bangladesh were also closely related to strains from Peru obtained in 2019 and 2021. The viruses most closely related to the Bangladesh cluster from 2017 to 2019 were Malaysia 2014 (MG895063) and Singapore 2013 (MW512387) (Figure 2). Further, the DENV2 cosmopolitan sequences from Bangladesh that were collected between 2004 and 2011 (12 sequences) and deposited in the NCBI database [36,37] grouped with clade B, which only persisted in India, Pakistan, Sri Lanka, Singapore, China, and Ethiopia. Whereas the Bangladesh 2017 strains showed the presence of two different clades, clade B and clade C, the present study failed to identify any clade B viruses in 2018, suggesting a complete shift from clade B to clade C in Bangladesh.

### 3.8. Phylogenetic Analysis of DENV3

**Figure 3:**
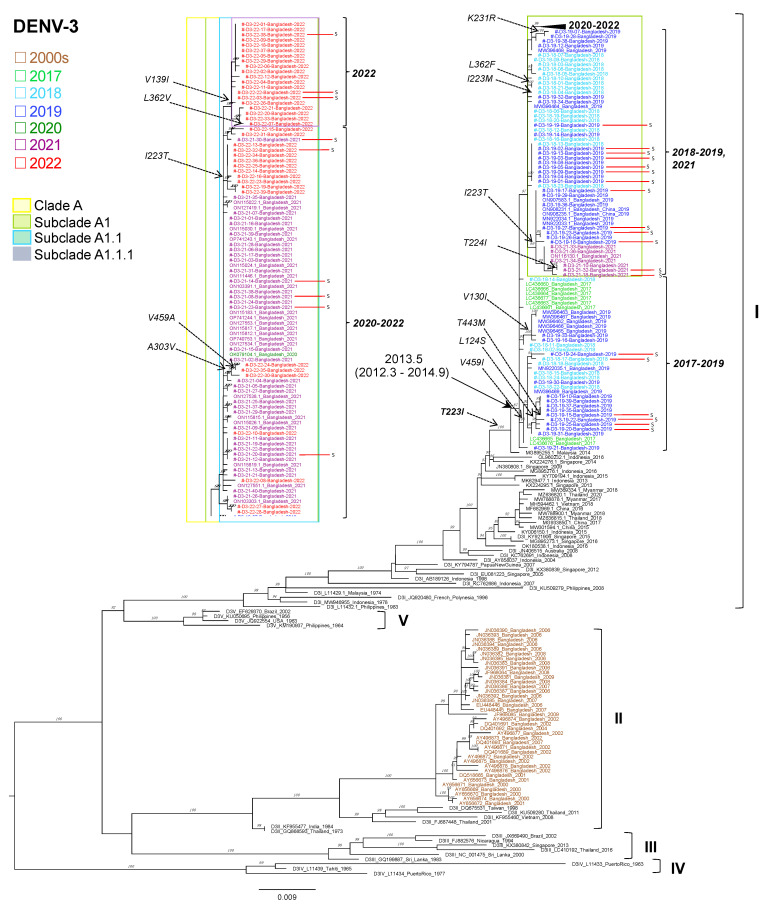
Genotyping of the DENV3 envelope region. A maximum likelihood phylogenetic tree was constructed using the W-IQ-TREE program in ModelFinder with ultrafast bootstrapping (UFBoot) and 1000 replicates. TN+ F+ G4 was selected as the best-fit model according to Bayesian information criteria. Data include DENV envelope-encoding sequences obtained in the present study (light blue for 2018, royal blue for 2019, violet for 2021, and red for 2022) along with sequences with known genotypes obtained from GenBank (green for 2017). The DENV sequences from Bangladesh from 2000 to 2009 are shown in brown. Viral genotypes are indicated to the right of sample names, which comprise the accession number, country, and reported year of each sequence. Clade/subclade are highlighted with color indicated in the legend. Numbers to the right of the branches are UFBoot support values exceeding 75%. Amino acid mutations are indicated adjacent to the key nodes. Sequences of the present study were marked as #. Red lines indicate the severe cases.

For the DENV3 samples, out of 402 DENV3 specimens, we determined the nucleotide sequences of the enveloped regions in 140 specimens (24 from 2018, 37 from 2019, 40 from 2021, and 39 from 2022). All of the samples in the present study clustered with DENV3 genotype I (Figure 3). The Bangladesh DENV3-I viruses were very closely related to strains collected in Bangladesh at a similar time. Viruses collected in Bangladesh from 2017 to 2022 formed a monophyletic clade (denoted as clade A), sharing a common ancestor with a 2014 Malaysian strain (MG895255). Furthermore, the DENV3-I virus was found to have circulated in Indonesia and Singapore in 2013–2016, and in Myanmar, Thailand, China, and Vietnam in 2015–2018 (Figure 3). The earliest DENV3 genotype I was identified in Malaysia in 1974 and Indonesia in 1978. Our previously published sequences for DENV3 in 2017 also clustered in the same genotype (genotype I), and these viruses are suspected to have been introduced to Bangladesh around mid-2013 (March 2012–September 2014) [35]. Although the sequences that were previously obtained for Bangladesh for 2000 to 2009 that were deposited in the NCBI database were grouped in with DENV3 genotype II, these genotype II viruses were not detected in the present study. This result suggests that the previously observed DENV3 genotype II in Bangladesh has been replaced by genotype I from 2017.

The mean number of nucleotide differences among DENV3 envelope regions, an indicator of genetic diversity of the virus, increased from 0.68 nucleotides in 2017 to 2.96 nucleotides in 2018 and 5.47 nucleotides in 2019. This value then decreased to 3.36 nucleotides in 2021, probably due to the effects of lockdown during the COVID-19 pandemic, before increasing again in 2022 to 4.45 nucleotides.

In Figure 3, the Bangladesh DENV3 genotype I viruses from 2018 to 2022 gradually evolved from Bangladesh 2017 and formed subclades within clade A in the order of subclades A1, A1.1, and A1.1.1. These clades and subclades were denoted in the present study. Regarding dengue severity, we found that 6 severe cases clustered in clade A during the period of 2018–2019 and another 13 cases clustered in subclade A.1 in 2019 and 2021. Furthermore, only six cases were reported in the period of 2021–2022 in A1.1 and three cases in 2022 in A.1.1.1. DENV3 patients with mild diseases were also distributed in subclades A.1, A.1.1, and A.1.1.1 of DENV3 genotype I, as with severe cases.

In 156 out of 179 DENV3 sequences that we sequenced, the amino acid at position 223 in the DENV3 envelope protein was occupied by isoleucine (223I). The closely related Malaysia 2014 strain also carried 223I, but all of the previous DENV3 genotype I sequences possessed threonine at position 223 (Figure 3). Therefore, this T223I substitution was considered to be characteristic of the current Bangladesh DENV3 genotype I sequences. However, an I-to-T reversion, I223T, was observed twice in our sequence dataset, once in 2019 in 13 sequences of subclade A1 and once in 2021 in 10 sequences of subclade A1.1 (Figure 3). Furthermore, two sequences with I223T reversion in subclade A1, i.e., D3-21-10 and D3-21-32, showed an additional substitution from threonine to isoleucine at the neighboring amino acid at position 224. In addition, a single sequence possessing methionine at amino position 223 (D3-18-04) was observed in another branch in subclade A1. These results suggest that the region around amino acid position 223 in the envelope protein of DENV3 genotype I may contain an antigenic epitope that can vary depending on the internal environment of infected individuals in Bangladesh.

## 4. Discussion

Dengue is endemic in Bangladesh and frequent outbreaks are a major public health concern. Several dengue serotypes and genotype changes have been observed in recent decades, and as reported in Nicaragua and Singapore, these changes have been directly associated with disease severity [38,39]. Since dengue is also endemic in the neighboring countries of India and Myanmar, there is always a risk of virus importation, the transmission of new serotypes, and an increased likelihood of genetic divergence. Increased travel, climate change, population growth, urbanization, and poor implementation of effective control measures have been responsible for serotype transmission and the subsequent public health consequences of these new strains [40]. Dengue virus infections show clinical manifestations that are common in other febrile illnesses, with laboratory findings including thrombocytopenia, hypoalbuminemia, and elevated liver enzymes [41]. This study focused on shifts in serotypes and genotypes, the phylogenetic relationships among strains, clinical manifestations, and hematologic/hepatic enzymatic markers associated with disease severity observed over five consecutive years (2018–2022).

Regarding the distribution of serotypes, DENV2 (40.9%) was prevalent in 2018, but DENV3 re-expanded (33.1%) in the same year. A similar study conducted by the Institute of Epidemiology, Disease Control and Research, Bangladesh, showed that the prevalence of DENV2, DENV3, and DENV1 was 41%, 31%, and 9%, respectively [42]. In 2019, DENV3 became the predominant serotype (91.9%) and DENV2 disappeared. In the 2019 dengue outbreak, about 100,000 cases were reported, which is more than twice the number of all cases reported in the previous two decades [43,44]. A large number of severe cases were recorded after the re-emergence of DENV3 due to waning DENV2 immunity cross-protection (i.e., the prolonged presence of DENV2 from 2003 to 2018) [38]. From 2020, DENV3 was the only serotype in circulation until August 2022 [45]. A single DENV3 serotype was identified in 2020, when the main focus shifted from febrile patients towards patients with COVID-19. For the last 50 years, the most prevalent serotype has been DENV2, but DENV3 and DENV4 were identified in some epidemics as well [46].

In the present study, to clarify the trends in the genotypic diversity of DENV in Bangladesh, we sequenced the DENV envelope region of 179 samples collected from 2018 to 2022 (2018; 59 samples, 2019; 41 samples, 2021; 40 samples; and 2022; 39 samples). Phylogenetic analysis of all nine DENV1 sequences identified clustered in genotype V, along with two GenBank sequences for Bangladesh from the 2000s, and six from 2017 that were analyzed by us previously [47]. Only four DENV1 envelope sequences from Bangladesh were deposited in the NCBI database before 2012, and these were also genotype V [48]. Evidence of DENV1 genotype V circulation was also reported for Bhutan, Nepal, Brazil, and Senegal [49,50,51,52]. In India, however, genotype V and I were co-circulating [53,54]. In Myanmar and Thailand, the most recent strain in circulation was DENV1 genotype I [33,55]. Since DENV1 was not detected in Bangladesh in 2020–2022, it would be important to know which genotype of DENV1 re-emerges in Bangladesh in the future.

Clade replacement of the DENV2 cosmopolitan genotype is an interesting finding of our study. Before 2017, DENV2 cosmopolitan clade B was the only genotype circulating in Bangladesh [36,37]. In a previous study on 2017 specimens, we reported the emergence of clade B viruses that were distantly related to the previous Bangladesh clade B viruses [47]. In the present study, all of the DENV2 samples clustered in cosmopolitan clade C, with the complete disappearance of clade B. A total of 30 samples from our study along with 46 GenBank sequences from Bangladesh (33 samples from 2017, 12 samples from 2004 to 2011, and 1 sample from 2019) were used to produce a phylogenetic tree. The recent emergence of DENV2 cosmopolitan clade C has also been reported in northeastern India, Thailand, and China [34,56,57]. It would be interesting to compare the in vitro growth characteristics of the clade B virus with those of the clade C virus to determine why clade B viruses disappeared from Bangladesh.

In the DENV3 phylogenetic tree, 140 sequences were included from our present study along with 82 previously published Bangladesh sequences (21 sequences from 2021, 1 from 2020, 14 from 2019, 8 from 2017, and 38 from the 2000s). All of the Bangladesh sequences clustered in genotype I, except the samples from the 2000s, which belonged to genotype II. We reported on the replacement of DENV3 genotype II by genotype I in 2017 [47], and we observed its persistence until 2022; it is possible that this genotype might have come to Bangladesh through Malaysia. DENV3 genotype I was found exclusively in Malaysia, Singapore, Indonesia, and China [58,59,60,61]. Frequent shifting and co-circulation of the DENV3 genotypes have been reported in Malaysia [60,61]. DENV3 genotype I was co-circulating with genotype III in Myanmar and Thailand [33,55]. Conversely, DENV3 genotype III was reported in Lucknow in India in 2013, and a multicenter study conducted in India in 2018 reported the same findings [53,62].

We compiled the clinical symptoms, complications, and hematologic and hepatic enzyme variations associated with severe cases in Bangladesh. Fever (100%) with pleural effusion (29.9%) and breathing difficulty (17.9%) was the most frequent reported symptom in severe dengue cases. We observed severe thrombocytopenia (59.7%), increased hematocrit (35.8%), hypoalbuminemia (83.6%), and elevated liver enzymes (86.6%) due to liver injury, which corroborates the findings of previous studies [41,63,64]. 

It has been reported that DENV4 is probably the least pathogenic serotype [65], but we did not observe DENV4 in Bangladesh. We then compared the disease severity with serotypes from 2018 to 2022. The re-emergence of DENV3 was observed, and it was shown to replace DENV2 as the dominant serotype. In 2019, DENV3 was the burgeoning serotype and there was no evidence of DENV2. More severe cases were observed in the 2019 outbreak of DENV3 [25]. It is possible that the weaker serotype-specific cross-reactivity between the re-emerged DENV3 and pre-existing major serotype DENV2 in 2013–2018 increased the severity of the disease. Severe dengue most commonly occurs among patients with secondary DENV infections, and cross-reactive antibody-dependent enhancement is supposedly responsible for the pathogenesis of severe dengue [66]. Therefore, reporting serotype and genotypic change early each year may help in predicting the disease’s severity and fatality. It also provides an opportunity for authorities to better prepare mass management procedures before seasonal outbreaks. Although we did not observe a 100% association between any particular virus sequence and severe diseases (Figure 3), among a cluster of eight 2019 DENV3 samples (D3-19-01, D3-19-02, D3-19-03, D3-19-04, D3-19-05, D3-19-08, D3-19-09, and D3-19-13) and one 2018 DENV3 sample (D3-18-23) in subclade A1, six were derived from severe cases (Figure 3). It is clear that further studies including whole-genome sequencing are necessary in order to identify factors responsible for the seemingly increased pathogenicity as well as the unprecedented explosive transmission of DENV3 genotype I in infected individuals in Bangladesh.

## Figures and Tables

**Table 1:** Demographic and hospitalization characteristics of patients at Evercare Hospital, 2018–2022.

	2018			2019		2020	2021	2022
Total number of tests (*n* = 3759)	1016			566		130	1171	876
Positive NS1 antigen/positive RT-PCR (*n* = 839)	174			132		30	315	188
Positive serotyping (*n* = 495)	127			86		1	178	103
Serotype	DENV1	DENV2	DENV3	DENV1	DENV3	DENV3	DENV3	DENV3
Positive cases	33 (26)	52 (40.9)	42 (33.1)	7 (8.1)	79 (91.9)	1 (100)	178 (100)	103 (100)
% male	8 (6.3)	12 (9.4)	13 (10.2)	2 (40)	31 (55.36)		19 (65.52)	11 (55)
Mean age	20	31	23	13	17		30	30
Children and adolescent	16 (12.6)	19 (15)	16 (12.6)	4 (4.7)	52 (60.5)	0	53 (29.8)	40 (38.8)
Adult	15 (11.8)	32 (25.2)	25 (19.7)	3 (3.5)	27 (31.4)	1 (100)	117 (65.7)	59 (57.3)
Elderly	2 (1.6)	1 (0.8)	1 (0.8)	0	0	0	8 (4.5)	4 (3.9)
Hospitalization								
Inpatients *n* (%)	13 (10.2)	29 (22.8)	22 (17.3)	5 (5.8)	64 (74.4)	0	34 (19.1)	21 (20.4)
Admission day	3 (1–5)	4 (1–6)	4 (1–7)	3 (1–4)	3 (1–9)		3 (1–4)	3 (1–4)
Days febrile	4 (2–6)	5 (2–8)	5 (2–8)	4 (2–6)	5 (2–8)		5 (2–8)	5 (2–8)
Days in hospital	5 (1–12)	5 (1–8)	5 (1–8)	3 (1–5)	5 (2–14)		5 (1–11)	5 (1–10)
Classical (%)	12 (92.3)	23 (79.3)	16 (72.7)	5 (100)	29 (45.3)		22 (64.7)	14 (66.7)
Severe (%)	1 (7.7)	6 (20.7)	6 (27.3)	0	35 (54.7)		12 (35.3)	7 (33.3)
Sequence Analysis (*n* = 179)	5	30	24	4	37		40	39

**Table 2:** Complications and symptoms of hospitalized patients with severe dengue.

Complications	Number of Patients, *n* = 67 (%) *
Bleeding	10 (14.9)
Pleural effusion	2 (29.9)
Breathlessness	12 (17.9)
Ascites	9 (13.4)
Oliguria	4 (6)
Hepatomegaly	4 (6)
Splenomegaly	2 (3)
Seizures	1 (1.5)
Multiple organ failure	3 (4.5)
Mechanical ventilation	3 (4.5)
Sepsis	3 (4.5)
Symptoms	
Anorexia	12 (17.9)
Nausea	36 (53.7)
Vomiting	36 (53.7)
Abdominal pain	37 (55.2)
Body ache	23 (34.3)
Headache	16 (23.9)
Cough	7 (10.4)
Diarrhea	6 (9)
Rash	9 (13.4)
Weakness	8 (11.9)

* Two patients died out of sixty-seven.

**Table 3:** Blood parameters obtained for hospitalized severe dengue patients.

Lab Parameters	No. of Patients, *n* = 67 (%)	Median Value	*p* Value *
Platelet count (/cumm)			
≤50,000	40 (59.7)	27,000	<0.001
>50,000	27 (40.3)	80,000	
Hematocrit value (%)			
Normal hematocrit (Male)	22 (32.8)	42	<0.001
Raised hematocrit (Male)	15 (22.4)	46.6	
Normal hematocrit (Female)	22 (32.8)	35.45	<0.001
Raised hematocrit (Female)	8 (11.9)	43.25	
Hemoglobin (g/dL)			
Normal (Male)	27 (40.3)	15.5	<0.001
Below normal (Male)	10 (14.9)	12.26	
Normal (Female)	10 (14.9)	14.75	<0.001
Below normal (Female)	20 (29.9)	11.4	
Albumin (g/dL)			
Hypoalbuminemia	56 (83.6)	2.9	<0.001
Normal	11 (16.4)	4	
Leukocyte count (/cumm)			
≤4000	16 (23.9)	2935	<0.001
>4000	51 (76.1)	6840	
Liver enzyme (IU/L)			
Raised ALT, AST	58 (86.6)	109.5, 123.5	<0.001 **
Normal ALT, AST	9 (13.4)	34, 30	<0.001 ***

* *p* value calculated with SPSS software using Kolmogorov–Smirnov test; platelet count: <50,000 considered as critical; hematocrit value: for males >44% and for females >40% considered as hemoconcentration indicator; hemoglobin value: normal range for males (13.8–17.2 g/dL) and for females (12.1–15.1 g/dL). Albumin: normal range (3.5–5.5 g/dL); liver enzyme, ALT: normal range: 0–44 IU/L. AST: normal range: 8–45. ** *p* value for ALT. *** *p* value for AST.

## Data Availability

The newly obtained sequences were deposited in GenBank with accession numbers OQ826841–OQ827019, as shown in Table S3. The viral sequences analyzed in the present study were retrieved from GenBank and BV-BRC and are listed in Table S2.

## Supplementary Materials

The following supporting information can be downloaded at: https://www.mdpi.com/article/10.3390/v15051144/s1. Table S1: primers used to amplify the envelope region and for sequencing; Table S2: GenBank sequences of the envelope region used for phylogenetic analysis; Table S3: newly determined sequences in the present study; Table S4: age and sex distribution of study population (serotype-positive); Table S5: severity of sequenced serotypes during the period 2018–2022.
