# Supplementary material for: Genetic Analysis of Dengue Virus in Severe and Non-Severe Cases in Dhaka, Bangladesh, in 2018–2022
Source: Viruses. 2023 May 10;15(5):1144. doi: 10.3390/v15051144 (PMC10222234; doi:10.3390/v15051144)
Supplement: Supplementary file 1 [file viruses-15-01144-s001.zip › viruses-2393404-supplementary.pdf]

Table S1. **Primers used to amplify the envelope region and for sequencing**

Amplification

Fragment		Antisense primers	Sequence 5'-3'	Sense-primer	Sequence 5'-3'
DENV1-E	One -step RT	d1a17	CCAATGGCYGCTGAYAGTCT	d1s1C	*GATGAGGGAAGATGGGG*AGTTGTTA
	Nested PCR	d1a17	CCAATGGCYGCTGAYAGTCT	d1s3	GTCTACGTGGAC ATAAACGTTCTGTCGCATTGGC
DENV2-E	One -step RT	2447R	TTCCAGCTCACAACGCAACCAC	d2s1C	*GATGAGGGAAGATGGGG*AGTTGTTA
	Nested PCR	2447R	TTCCAGCTCACAACGCAACCAC	618F	GTCTACGTGGAC ACCAGAAGACATAGAYTGTTGGTG
DENV3-E	One -step RT	d3s2	CAACATGTGCACACTCATAGCC	DV3_R	CCACAACTACCGTTAATTTGAT
	Nested PCR	d3_815F	GCCCTTAGGCACCCAGGGTT	DV3_R	CCACAACTACCGTTAATTTGAT

Sequencing

Fragment	Sense-primer	Sequence 5'-3'
DENV1-E	1471R	TCCGTAGTCRGTCAGCT
	d1s4(1204F)	TGTGTGTCGMCGAACGTT
	d1s5(1709F)	GCAATGCAYACTGCGTTG
	d1a17(2559R)	CCRATGGCYGCTGAYAGTCT
DENV2-E	865Fx	ACCATAGGRACRACAYATTTCC
	1222F	ATGGTAGAYAGAGGATGGG
	1577Fx	TAGACCTGCCRTTACCATG
	2037F	AGAAGCAGAACCYCCATTYG
DENV3-E	815F	GCCCTTAGGCACCCAGGGTT
	1243F	CGGTTGTGGTTTGTTTGG
	1531F	GAAGAACAAAGCATGGATGGTA
	2590R	CCACAACTACCGTTAATTTGAT

**Table S2.** GenBank sequences of the envelope region used for phylogenetic analysis				
**Serotype**	**Genotype**	**Accession Number**	**Country**	**Year**
DENV-1	I	KX595191	Vietnam	2013
DENV-1	I	KF887994	Thailand	2013
DENV-1	I	HG316481	Thailand	2010
DENV-1	I	AY732482	Thailand	2001
DENV-1	I	AY732475	Thailand	1994
DENV-1	I	AY732480	Thailand	2004
DENV-1	I	AY732477	Thailand	1991
DENV-1	I	AF350498	China	1980
DENV-1	I	AY732481	Thailand	1982
DENV-1	I	AY732483	Thailand	1981
DENV-1	I	AB074760	Japan	1943
DENV-1	II	AF180817	Thailand	1964
DENV-1	III	EF457905	Malaysia	1972
DENV-1	IV	DQ672560	French Polynesia	2001
DENV-1	IV	AB189120	Indonesia	1998
DENV-1	IV	DQ285560	Reunion	2004
DENV-1	IV	KC762651	Indonesia	2007
DENV-1	IV	GQ868602	Philippines	2004
DENV-1	V	MK517747	China	2018
DENV-1	V	MK517746	Bangladesh	2017
DENV-1	V	MK517743	China	2018
DENV-1	V	MG894942	Maldives	2016
DENV-1	V	LC436668	Bangladesh	2017
DENV-1	V	LC436610	Bangladesh	2017
DENV-1	V	LC436667	Bangladesh	2017
DENV-1	V	MG738056	China	2016
DENV-1	V	MG894946	Malaysia	2016
DENV-1	V	MG894912	Maldives	2016
DENV-1	V	MG894884	Thailand	2015
DENV-1	V	LC436608	Bangladesh	2017
DENV-1	V	MG737898	Bangladesh	2015
DENV-1	V	MG894789	Malaysia	2013
DENV-1	V	LC038145	Malaysia	2014
DENV-1	V	LC436612	Bangladesh	2017
DENV-1	V	LC436607	Bangladesh	2017
DENV-1	V	LC436609	Bangladesh	2017
DENV-1	V	MG840568	China	2016
DENV-1	V	MG738024	China	2016
DENV-1	V	MF033243	Singapore	2016
DENV-1	V	MH680025	Singapore	2014
DENV-1	V	KM403585	Singapore	2013
DENV-1	V	MG894690	Bangladesh	2011

DENV-1	V	KP849889	Bhutan	2013
DENV-1	V	KP849864	Bhutan	2013
DENV-1	V	MH679963	Singapore	2015
DENV-1	V	KX380803	Singapore	2013
DENV-1	V	MG894721	Bangladesh	2012
DENV-1	V	MG894720	Bangladesh	2012
DENV-1	V	MH680112	Singapore	2014
DENV-1	V	MH679981	Singapore	2015
DENV-1	V	KX380801	Singapore	2012
DENV-1	V	KP723473	China	2014
DENV-1	V	KX621249	China	2014
DENV-1	V	JN036371	Bangladesh	2009
DENV-1	V	KF289072	India	2011
DENV-1	V	JQ917404	India	2009
DENV-1	V	AY732474	Thailand	1980
DENV-1	V	AY732476	Thailand	1980
DENV-1	V	AY722803	Myanmar	1998
DENV-1	V	AF226685	Brazil	1990
DENV-1	V	AF514889	Argentina	2000
DENV-1	VI	KR919820	Brunei	2014
DENV-2	Cosmopolitan C	OM791801.1	Peru	2019
DENV-2	Cosmopolitan C	ON123639.1	Peru	2021
DENV-2	Cosmopolitan C	ON123643.1	Peru	2021
DENV-2	Cosmopolitan C	ON123642.1	Peru	2021
DENV-2	Cosmopolitan C	ON123638.1	Peru	2021
DENV-2	Cosmopolitan C	MK848418.1	Bangladesh	2018
DENV-2	Cosmopolitan C	LC436620.1	Bangladesh	2017
DENV-2	Cosmopolitan C	LC436672	Bangladesh	2017
DENV-2	Cosmopolitan C	LC436653.1	Bangladesh	2017
DENV-2	Cosmopolitan C	LC436617.1	Bangladesh	2017
DENV-2	Cosmopolitan C	LC436646	Bangladesh	2017
DENV-2	Cosmopolitan C	LC436652	Bangladesh	2017
DENV-2	Cosmopolitan C	LC436651	Bangladesh	2017
DENV-2	Cosmopolitan C	MN328061.1	Bangladesh	2019
DENV-2	Cosmopolitan C	LC436627	Bangladesh	2017
DENV-2	Cosmopolitan C	LC436640	Bangladesh	2017
DENV-2	Cosmopolitan C	LC436632	Bangladesh	2017
DENV-2	Cosmopolitan C	LC436633	Bangladesh	2017
DENV-2	Cosmopolitan C	LC436645	Bangladesh	2017
DENV-2	Cosmopolitan C	LC436630	Bangladesh	2017
DENV-2	Cosmopolitan C	LC43662909	Bangladesh	2017
DENV-2	Cosmopolitan C	LC436624	Bangladesh	2017
DENV-2	Cosmopolitan C	LC436644	Bangladesh	2017
DENV-2	Cosmopolitan C	LC436657	Bangladesh	2017
DENV-2	Cosmopolitan C	LC436648	Bangladesh	2017
DENV-2	Cosmopolitan C	LC436637	Bangladesh	2017
DENV-2	Cosmopolitan C	LC436634	Bangladesh	2017
DENV-2	Cosmopolitan C	LC436638	Bangladesh	2017
DENV-2	Cosmopolitan C	LC436613	Bangladesh	2017

DENV-2	Cosmopolitan C	LC436669	Bangladesh	2017
DENV-2	Cosmopolitan C	LC436649	Bangladesh	2017
DENV-2	Cosmopolitan C	LC436642	Bangladesh	2017
DENV-2	Cosmopolitan C	LC436623	Bangladesh	2017
DENV-2	Cosmopolitan C	LC436641	Bangladesh	2017
DENV-2	Cosmopolitan C	LC436626	Bangladesh	2017
DENV-2	Cosmopolitan C	LC436635	Bangladesh	2017
DENV-2	Cosmopolitan C	LC436636	Bangladesh	2017
DENV-2	Cosmopolitan C	LC436622	Bangladesh	2017
DENV-2	Cosmopolitan C	LC436628	Bangladesh	2017
DENV-2	Cosmopolitan C	LC436631.1	Bangladesh	2017
DENV-2	Cosmopolitan C	LC436639	Bangladesh	2017
DENV-2	Cosmopolitan C	LC436625.1	Bangladesh	2017
DENV-2	Cosmopolitan C	LC436643.1	Bangladesh	2017
DENV-2	Cosmopolitan C	MG895063.1	Malaysia	2014
DENV-2	Cosmopolitan C	LC436614	Bangladesh	2017
DENV-2	Cosmopolitan C	LC436675	Bangladesh	2017
DENV-2	Cosmopolitan C	LC436659	Bangladesh	2017
DENV-2	Cosmopolitan C	LC436615	Bangladesh	2017
DENV-2	Cosmopolitan C	LC436674	Bangladesh	2017
DENV-2	Cosmopolitan C	LC436656	Bangladesh	2017
DENV-2	Cosmopolitan C	LC436647	Bangladesh	2017
DENV-2	Cosmopolitan C	LC436616	Bangladesh	2017
DENV-2	Cosmopolitan C	LC436673	Bangladesh	2017
DENV-2	Cosmopolitan C	LC436655	Bangladesh	2017
DENV-2	Cosmopolitan C	LC436654	Bangladesh	2017
DENV-2	Cosmopolitan C	LC436658	Bangladesh	2017
DENV-2	Cosmopolitan C	MW512387.1	Singapore	2013
DENV-2	Cosmopolitan C	MN955685	Thailand	2018
DENV-2	Cosmopolitan C	LC410191	Thailand	2017
DENV-2	Cosmopolitan C	LC410190	Thailand	2016
DENV-2	Cosmopolitan C	MZ277524	India	2018
DENV-2	Cosmopolitan C	KX621247	China	2015
DENV-2	Cosmopolitan C	KX372564.1	Australia	2015
DENV-2	Cosmopolitan C	KY921905.1	Singapore	2015
DENV-2	Cosmopolitan C	MW512457.1	Singapore	2016
DENV-2	Cosmopolitan C	KY495802	India	2016
DENV-2	Cosmopolitan C	MN602605.1	Sri Lanka	2017
DENV-2	Cosmopolitan C	MG895089.1	Malaysia	2015
DENV-2	Cosmopolitan C	KU517847.1	Philippines	2015
DENV-2	Cosmopolitan C	KX452030	Malaysia	2014
DENV-2	Cosmopolitan C	MW512395.1	Singapore	2013
DENV-2	Cosmopolitan C	KT232052.1	Nepal	2013
DENV-2	Cosmopolitan C	JF327392	Singapore	2009
DENV-2	Cosmopolitan C	EU179857	Brunei	2005
DENV-2	Cosmopolitan C	HM488257	Guam	2001
DENV-2	Cosmopolitan C	AY858035	Indonesia	2004
DENV-2	Cosmopolitan C	EU482672	Vietnam	2006
DENV-2	Cosmopolitan C	EU081177	Singapore	2005

DENV-2	Cosmopolitan C	KY794785	Papua New Guinea	2010
DENV-2	Cosmopolitan C	AY037116	Australia	1993
DENV-2	Cosmopolitan C	FJ196853	China	2003
DENV-2	Cosmopolitan C	AB189122	Indonesia	1998
DENV-2	Cosmopolitan C	DQ645546	Taiwan	2002
DENV-2	Cosmopolitan A	EU056810	Burkina Faso	1983
DENV-2	Cosmopolitan A	L10044.1	Indonesia	1976
DENV-2	Cosmopolitan A	GQ398259	Indonesia	1976
DENV-2	Cosmopolitan A	GQ398258	Indonesia	1975
DENV-2	Cosmopolitan B	KT781532	Bangladesh	2011
DENV-2	Cosmopolitan B	JN036375	Bangladesh	2008
DENV-2	Cosmopolitan B	JN036372	Bangladesh	2009
DENV-2	Cosmopolitan B	JN036379	Bangladesh	2006
DENV-2	Cosmopolitan B	JN036377	Bangladesh	2008
DENV-2	Cosmopolitan B	JN036380	Bangladesh	2006
DENV-2	Cosmopolitan B	FJ898454	India	2006
DENV-2	Cosmopolitan B	JN036378	Bangladesh	2007
DENV-2	Cosmopolitan B	JN036374	Bangladesh	2009
DENV-2	Cosmopolitan B	JN036373	Bangladesh	2009
DENV-2	Cosmopolitan B	JN036376	Bangladesh	2008
DENV-2	Cosmopolitan B	EU448423	Bangladesh	2005
DENV-2	Cosmopolitan B	EU448424	Bangladesh	2004
DENV-2	Cosmopolitan B	MZ277456	India	2018
DENV-2	Cosmopolitan B	MH594921	India	2017
DENV-2	Cosmopolitan B	MK858105	India	2016
DENV-2	Cosmopolitan B	KF364499	India	2012
DENV-2	Cosmopolitan B	KF364504	India	2008
DENV-2	Cosmopolitan B	KF577806	India	2010
DENV-2	Cosmopolitan B	JQ955624	India	2011
DENV-2	Cosmopolitan B	GQ252677	Sri Lanka	2004
DENV-2	Cosmopolitan B	KJ010186	Pakistan	2013
DENV-2	Cosmopolitan B	AF276619	China	2000
DENV-2	Cosmopolitan B	AF359579	China	1999
DENV-2	Cosmopolitan B	LC436618	Bangladesh	2017
DENV-2	Cosmopolitan B	LC436671	Bangladesh	2017
DENV-2	Cosmopolitan B	LC436650	Bangladesh	2017
DENV-2	Cosmopolitan B	LC436619	Bangladesh	2017
DENV-2	Cosmopolitan B	LC436670	Bangladesh	2017
DENV-2	Cosmopolitan B	MW512449.1	Singapore	2016
DENV-2	Cosmopolitan B	MH822952.1	India	2014
DENV-2	Cosmopolitan B	MH209618.1	India	2015
DENV-2	Cosmopolitan B	MK858096.1	India	2014
DENV-2	Cosmopolitan B	KX061419.1	India	2015
DENV-2	Cosmopolitan B	LC121816	Ethiopia	2016
DENV-2	Cosmopolitan B	JX475906	India	2009
DENV-2	Cosmopolitan B	MZ277472	India	2018
DENV-2	Cosmopolitan B	KY427084	India	2010
DENV-2	Cosmopolitan B	FJ882602	Sri Lanka	1996
DENV-2	Cosmopolitan B	MW946478	India	1974

DENV-2	Cosmopolitan B	AF231716.1	Malaysia	1969
DENV-2	Asian-I	FJ898452	Thailand	2003
DENV-2	Asian-I	MS8455	Malaysia	1987
DENV-2	Asian-I	D00345.1	Thailand	1980
DENV-2	Asian-I	NC001474	Thailand	1964
DENV-2	Asian-II	AF038403	New Guinea	1944
DENV-2	Asian-II	GQ398268	Indonesia	1975
DENV-2	Asian/ American	HQ999999	Guatemala-	2009
DENV-2	Asian/ American	KF955363.1	Puerto Rico	1986
DENV-2	Asian/ American	JN819418.1	Vietnam	1988
DENV-2	American	GQ868592	Colombia	1986
DENV-3	I	MW396468	Bangladesh	2019
DENV-3	I	MW396464	Bangladesh	2019
DENV-3	I	ON907583.1	Bangladesh	2019
DENV-3	I	ON908231.1	Bangladesh China	2019
DENV-3	I	ON908235.1	Bangladesh China	2019
DENV-3	I	MN922034.1	Bangladesh	2019
DENV-3	I	MN922033.1	Bangladesh	2019
DENV-3	I	ON116130.1	Bangladesh	2021
DENV-3	I	LC436660	Bangladesh	2017
DENV-3	I	LC436666	Bangladesh	2017
DENV-3	I	LC436664	Bangladesh	2017
DENV-3	I	LC436677	Bangladesh	2017
DENV-3	I	LC436663	Bangladesh	2017
DENV-3	I	LC436661	Bangladesh	2017
DENV-3	I	MW396463	Bangladesh	2019
DENV-3	I	MW396467	Bangladesh	2019
DENV-3	I	MW396462	Bangladesh	2019
DENV-3	I	MW396466	Bangladesh	2019
DENV-3	I	MW396465	Bangladesh	2019
DENV-3	I	MN922035.1	Bangladesh	2019
DENV-3	I	MW396469	Bangladesh	2019
DENV-3	I	LC436665	Bangladesh	2017
DENV-3	I	LC436676	Bangladesh	2017
DENV-3	I	MG895255.1	Malaysia	2014
DENV-3	I	OL960232.1	Indonesia	2016
DENV-3	I	KX224276.1	Singapore	2014
DENV-3	I	JN380808.1	Singapore	2009
DENV-3	I	MG895276.1	Indonesia	2016
DENV-3	I	KY709194.1	Indonesia	2015
DENV-3	I	MK629477.1	Indonesia	2013
DENV-3	I	KX224295.1	Singapore	2013
DENV-3	I	MW369334.1	Myanmar	2018
DENV-3	I	MZ636820.1	Thailand	2020
DENV-3	I	MW788878.1	Myanmar	2017
DENV-3	I	MH594462.1	Vietnam	2018
DENV-3	I	MF682969.1	China	2016
DENV-3	I	MW788900.1	Myanmar	2018
DENV-3	I	MZ636815.1	Thailand	2018

DENV-3	I	MG933850.1	China	2017
DENV-3	I	MW301594.1	China	2015
DENV-3	I	KY006150.1	Indonesia	2015
DENV-3	I	KY921906	Singapore	2015
DENV-3	I	MG895273.1	Singapore	2016
DENV-3	I	OK180538.1	Indonesia	2016
DENV-3	I	JN406515	Australia	2008
DENV-3	I	KC762691	Indonesia	2008
DENV-3	I	AY858037	Indonesia	2004
DENV-3	I	KY794787	Papua New Guinea	2007
DENV-3	I	KX380839	Singapore	2012
DENV-3	I	EU081223	Singapore	2005
DENV-3	I	AB189126	Indonesia	1998
DENV-3	I	KC762686	Indonesia	2007
DENV-3	I	KU509279	Philippines	2008
DENV-3	I	L11429.1	Malaysia	1974
DENV-3	I	JQ920480	French Polynesia	1996
DENV-3	I	MW946955	Indonesia	1978
DENV-3	I	L11432.1	Philippines	1983
DENV-3	I	ON115022.1	Bangladesh	2021
DENV-3	I	ON127419.1	Bangladesh	2021
DENV-3	I	ON115030.1	Bangladesh	2021
DENV-3	I	OP741243.1	Bangladesh	2021
DENV-3	I	ON115024.1	Bangladesh	2021
DENV-3	I	ON111446.1	Bangladesh	2021
DENV-3	I	ON103391.1	Bangladesh	2021
DENV-3	I	ON115183.1	Bangladesh	2021
DENV-3	I	OP741244.1	Bangladesh	2021
DENV-3	I	ON127553.1	Bangladesh	2021
DENV-3	I	ON115817.1	Bangladesh	2021
DENV-3	I	ON115812.1	Bangladesh	2021
DENV-3	I	OP740753.1	Bangladesh	2021
DENV-3	I	ON127534.1	Bangladesh	2021
DENV-3	I	OK079104.1	Bangladesh	2020
DENV-3	I	ON127538.1	Bangladesh	2021
DENV-3	I	ON115815.1	Bangladesh	2021
DENV-3	I	ON115026.1	Bangladesh	2021
DENV-3	I	ON115819.1	Bangladesh	2021
DENV-3	I	ON127551.1	Bangladesh	2021
DENV-3	I	ON103303.1	Bangladesh	2021
DENV-3	II	JN036390	Bangladesh	2006
DENV-3	II	JN036393	Bangladesh	2006
DENV-3	II	JN036388	Bangladesh	2006
DENV-3	II	JN036394	Bangladesh	2006
DENV-3	II	JN036389	Bangladesh	2006
DENV-3	II	JN036382	Bangladesh	2008
DENV-3	II	JN036395	Bangladesh	2006
DENV-3	II	JN036383	Bangladesh	2008
DENV-3	II	JN036391	Bangladesh	2006

DENV-3	II	JF968064	Bangladesh	2008
DENV-3	II	JN036381	Bangladesh	2009
DENV-3	II	JN036384	Bangladesh	2008
DENV-3	II	JN036386	Bangladesh	2007
DENV-3	II	JN036387	Bangladesh	2006
DENV-3	II	JN036392	Bangladesh	2006
DENV-3	II	JN036385	Bangladesh	2007
DENV-3	II	EU448446	Bangladesh	2006
DENV-3	II	EU448445	Bangladesh	2007
DENV-3	II	JF968085	Bangladesh	2009
DENV-3	II	AY496874	Bangladesh	2002
DENV-3	II	DQ401691	Bangladesh	2002
DENV-3	II	DQ401692	Bangladesh	2004
DENV-3	II	AY496877	Bangladesh	2002
DENV-3	II	AY496873	Bangladesh	2002
DENV-3	II	DQ401693	Bangladesh	2007
DENV-3	II	AY496871	Bangladesh	2002
DENV-3	II	DQ401689	Bangladesh	2002
DENV-3	II	AY496872	Bangladesh	2002
DENV-3	II	AY496875	Bangladesh	2002
DENV-3	II	AY496878	Bangladesh	2002
DENV-3	II	AY496876	Bangladesh	2002
DENV-3	II	DQ518665	Bangladesh	2001
DENV-3	II	AY656673	Bangladesh	2001
DENV-3	II	AY656671	Bangladesh	2000
DENV-3	II	AY656669	Bangladesh	2000
DENV-3	II	AY656670	Bangladesh	2000
DENV-3	II	AY656674	Bangladesh	2000
DENV-3	II	AY656672	Bangladesh	2001
DENV-3	II	DQ675531	Taiwan	1998
DENV-3	II	KU509280	Thailand	2011
DENV-3	II	KF955460	Vietnam	2008
DENV-3	II	FJ687448	Thailand	2001
DENV-3	II	KF955477	India	1984
DENV-3	II	GQ868593	Thailand	1973
DENV-3	III	JX669490	Brazil	2002
DENV-3	III	FJ882576	Nicaragua	1994
DENV-3	III	KX380842	Singapore	2013
DENV-3	III	LC410192	Thailand	2016
DENV-3	III	NC_001475	Sri Lanka	2000
DENV-3	III	GQ199887	Sri Lanka	1983
DENV-3	IV	L11433	Puerto Rico	1963
DENV-3	IV	L11439	Tahiti	1965
DENV-3	IV	L11434	Puerto Rico	1977
DENV-3	V	EF629370	Brazil	2002
DENV-3	V	KU050695	Philippines	1956
DENV-3	V	JQ922554	USA	1963
DENV-3	V	KM190937	Philippines	1964

Table S3. Newly determined Sequences in the present study

Serotype	Genotype	Sequence ID	Accession Number	Country	Year
DENV-1	V	D1-18-01	OQ826841	Bangladesh	2018
DENV-1	V	D1-18-02	OQ826842	Bangladesh	2018
DENV-1	V	D1-18-03	OQ826843	Bangladesh	2018
DENV-1	V	D1-18-04	OQ826844	Bangladesh	2018
DENV-1	V	D1-18-05	OQ826845	Bangladesh	2018
DENV-1	V	D1-19-06	OQ826846	Bangladesh	2019
DENV-1	V	D1-19-07	OQ826847	Bangladesh	2019
DENV-1	V	D1-19-08	OQ826848	Bangladesh	2019
DENV-1	V	D1-19-09	OQ826849	Bangladesh	2019
DENV-2	Cosmopolitan C	D2-18-01	OQ826850	Bangladesh	2018
DENV-2	Cosmopolitan C	D2-18-02	OQ826851	Bangladesh	2018
DENV-2	Cosmopolitan C	D2-18-03	OQ826852	Bangladesh	2018
DENV-2	Cosmopolitan C	D2-18-04	OQ826853	Bangladesh	2018
DENV-2	Cosmopolitan C	D2-18-05	OQ826854	Bangladesh	2018
DENV-2	Cosmopolitan C	D2-18-06	OQ826855	Bangladesh	2018
DENV-2	Cosmopolitan C	D2-18-07	OQ826856	Bangladesh	2018
DENV-2	Cosmopolitan C	D2-18-08	OQ826857	Bangladesh	2018
DENV-2	Cosmopolitan C	D2-18-09	OQ826858	Bangladesh	2018
DENV-2	Cosmopolitan C	D2-18-10	OQ826859	Bangladesh	2018
DENV-2	Cosmopolitan C	D2-18-11	OQ826860	Bangladesh	2018
DENV-2	Cosmopolitan C	D2-18-12	OQ826861	Bangladesh	2018
DENV-2	Cosmopolitan C	D2-18-13	OQ826862	Bangladesh	2018
DENV-2	Cosmopolitan C	D2-18-14	OQ826863	Bangladesh	2018
DENV-2	Cosmopolitan C	D2-18-15	OQ826864	Bangladesh	2018
DENV-2	Cosmopolitan C	D2-18-16	OQ826865	Bangladesh	2018
DENV-2	Cosmopolitan C	D2-18-17	OQ826866	Bangladesh	2018
DENV-2	Cosmopolitan C	D2-18-19	OQ826867	Bangladesh	2018
DENV-2	Cosmopolitan C	D2-18-20	OQ826868	Bangladesh	2018
DENV-2	Cosmopolitan C	D2-18-21	OQ826869	Bangladesh	2018
DENV-2	Cosmopolitan C	D2-18-23	OQ826870	Bangladesh	2018
DENV-2	Cosmopolitan C	D2-18-24	OQ826871	Bangladesh	2018
DENV-2	Cosmopolitan C	D2-18-25	OQ826872	Bangladesh	2018
DENV-2	Cosmopolitan C	D2-18-27	OQ826873	Bangladesh	2018
DENV-2	Cosmopolitan C	D2-18-28	OQ826874	Bangladesh	2018
DENV-2	Cosmopolitan C	D2-18-29	OQ826875	Bangladesh	2018
DENV-2	Cosmopolitan C	D2-18-31	OQ826876	Bangladesh	2018
DENV-2	Cosmopolitan C	D2-18-32	OQ826877	Bangladesh	2018
DENV-2	Cosmopolitan C	D2-18-33	OQ826878	Bangladesh	2018
DENV-2	Cosmopolitan C	D2-18-35	OQ826879	Bangladesh	2018

DENV-3	I	D3-18-01	OQ826880	Bangladesh	2018
DENV-3	I	D3-18-02	OQ826881	Bangladesh	2018
DENV-3	I	D3-18-03	OQ826882	Bangladesh	2018
DENV-3	I	D3-18-04	OQ826883	Bangladesh	2018
DENV-3	I	D3-18-05	OQ826884	Bangladesh	2018
DENV-3	I	D3-18-06	OQ826885	Bangladesh	2018
DENV-3	I	D3-18-07	OQ826886	Bangladesh	2018
DENV-3	I	D3-18-08	OQ826887	Bangladesh	2018
DENV-3	I	D3-18-09	OQ826888	Bangladesh	2018
DENV-3	I	D3-18-10	OQ826889	Bangladesh	2018
DENV-3	I	D3-18-11	OQ826890	Bangladesh	2018
DENV-3	I	D3-18-12	OQ826891	Bangladesh	2018
DENV-3	I	D3-18-13	OQ826892	Bangladesh	2018
DENV-3	I	D3-18-14	OQ826893	Bangladesh	2018
DENV-3	I	D3-18-15	OQ826894	Bangladesh	2018
DENV-3	I	D3-18-16	OQ826895	Bangladesh	2018
DENV-3	I	D3-18-17	OQ826896	Bangladesh	2018
DENV-3	I	D3-18-18	OQ826897	Bangladesh	2018
DENV-3	I	D3-18-19	OQ826898	Bangladesh	2018
DENV-3	I	D3-18-20	OQ826899	Bangladesh	2018
DENV-3	I	D3-18-21	OQ826900	Bangladesh	2018
DENV-3	I	D3-18-22	OQ826901	Bangladesh	2018
DENV-3	I	D3-18-23	OQ826902	Bangladesh	2018
DENV-3	I	D3-18-24	OQ826903	Bangladesh	2018
DENV-3	I	D3-19-01	OQ826904	Bangladesh	2019
DENV-3	I	D3-19-02	OQ826905	Bangladesh	2019
DENV-3	I	D3-19-03	OQ826906	Bangladesh	2019
DENV-3	I	D3-19-04	OQ826907	Bangladesh	2019
DENV-3	I	D3-19-05	OQ826908	Bangladesh	2019
DENV-3	I	D3-19-07	OQ826909	Bangladesh	2019
DENV-3	I	D3-19-08	OQ826910	Bangladesh	2019
DENV-3	I	D3-19-09	OQ826911	Bangladesh	2019
DENV-3	I	D3-19-10	OQ826912	Bangladesh	2019
DENV-3	I	D3-19-12	OQ826913	Bangladesh	2019
DENV-3	I	D3-19-13	OQ826914	Bangladesh	2019
DENV-3	I	D3-19-14	OQ826915	Bangladesh	2019
DENV-3	I	D3-19-15	OQ826916	Bangladesh	2019
DENV-3	I	D3-19-16	OQ826917	Bangladesh	2019
DENV-3	I	D3-19-17	OQ826918	Bangladesh	2019
DENV-3	I	D3-19-18	OQ826919	Bangladesh	2019
DENV-3	I	D3-19-19	OQ826920	Bangladesh	2019

DENV-3	I	D3-19-20	OQ826921	Bangladesh	2019
DENV-3	I	D3-19-21	OQ826922	Bangladesh	2019
DENV-3	I	D3-19-22	OQ826923	Bangladesh	2019
DENV-3	I	D3-19-23	OQ826924	Bangladesh	2019
DENV-3	I	D3-19-24	OQ826925	Bangladesh	2019
DENV-3	I	D3-19-25	OQ826926	Bangladesh	2019
DENV-3	I	D3-19-26	OQ826927	Bangladesh	2019
DENV-3	I	D3-19-27	OQ826928	Bangladesh	2019
DENV-3	I	D3-19-28	OQ826929	Bangladesh	2019
DENV-3	I	D3-19-29	OQ826930	Bangladesh	2019
DENV-3	I	D3-19-30	OQ826931	Bangladesh	2019
DENV-3	I	D3-19-31	OQ826932	Bangladesh	2019
DENV-3	I	D3-19-32	OQ826933	Bangladesh	2019
DENV-3	I	D3-19-33	OQ826934	Bangladesh	2019
DENV-3	I	D3-19-34	OQ826935	Bangladesh	2019
DENV-3	I	D3-19-35	OQ826936	Bangladesh	2019
DENV-3	I	D3-19-36	OQ826937	Bangladesh	2019
DENV-3	I	D3-19-37	OQ826938	Bangladesh	2019
DENV-3	I	D3-19-38	OQ826939	Bangladesh	2019
DENV-3	I	D3-19-39	OQ826940	Bangladesh	2019
DENV-3	I	D3-21-01	OQ826941	Bangladesh	2021
DENV-3	I	D3-21-02	OQ826942	Bangladesh	2021
DENV-3	I	D3-21-03	OQ826943	Bangladesh	2021
DENV-3	I	D3-21-04	OQ826944	Bangladesh	2021
DENV-3	I	D3-21-05	OQ826945	Bangladesh	2021
DENV-3	I	D3-21-06	OQ826946	Bangladesh	2021
DENV-3	I	D3-21-07	OQ826947	Bangladesh	2021
DENV-3	I	D3-21-08	OQ826948	Bangladesh	2021
DENV-3	I	D3-21-09	OQ826949	Bangladesh	2021
DENV-3	I	D3-21-10	OQ826950	Bangladesh	2021
DENV-3	I	D3-21-11	OQ826951	Bangladesh	2021
DENV-3	I	D3-21-12	OQ826952	Bangladesh	2021
DENV-3	I	D3-21-13	OQ826953	Bangladesh	2021
DENV-3	I	D3-21-14	OQ826954	Bangladesh	2021
DENV-3	I	D3-21-15	OQ826955	Bangladesh	2021
DENV-3	I	D3-21-16	OQ826956	Bangladesh	2021
DENV-3	I	D3-21-17	OQ826957	Bangladesh	2021
DENV-3	I	D3-21-18	OQ826958	Bangladesh	2021
DENV-3	I	D3-21-19	OQ826959	Bangladesh	2021
DENV-3	I	D3-21-20	OQ826960	Bangladesh	2021
DENV-3	I	D3-21-21	OQ826961	Bangladesh	2021

DENV-3	I	D3-21-22	OQ826962	Bangladesh	2021
DENV-3	I	D3-21-23	OQ826963	Bangladesh	2021
DENV-3	I	D3-21-24	OQ826964	Bangladesh	2021
DENV-3	I	D3-21-25	OQ826965	Bangladesh	2021
DENV-3	I	D3-21-26	OQ826966	Bangladesh	2021
DENV-3	I	D3-21-27	OQ826967	Bangladesh	2021
DENV-3	I	D3-21-28	OQ826968	Bangladesh	2021
DENV-3	I	D3-21-29	OQ826969	Bangladesh	2021
DENV-3	I	D3-21-30	OQ826970	Bangladesh	2021
DENV-3	I	D3-21-31	OQ826971	Bangladesh	2021
DENV-3	I	D3-21-32	OQ826972	Bangladesh	2021
DENV-3	I	D3-21-33	OQ826973	Bangladesh	2021
DENV-3	I	D3-21-34	OQ826974	Bangladesh	2021
DENV-3	I	D3-21-35	OQ826975	Bangladesh	2021
DENV-3	I	D3-21-36	OQ826976	Bangladesh	2021
DENV-3	I	D3-21-37	OQ826977	Bangladesh	2021
DENV-3	I	D3-21-38	OQ826978	Bangladesh	2021
DENV-3	I	D3-21-39	OQ826979	Bangladesh	2021
DENV-3	I	D3-21-40	OQ826980	Bangladesh	2021
DENV-3	I	D3-22-01	OQ826981	Bangladesh	2022
DENV-3	I	D3-22-02	OQ826982	Bangladesh	2022
DENV-3	I	D3-22-03	OQ826983	Bangladesh	2022
DENV-3	I	D3-22-04	OQ826984	Bangladesh	2022
DENV-3	I	D3-22-05	OQ826985	Bangladesh	2022
DENV-3	I	D3-22-06	OQ826986	Bangladesh	2022
DENV-3	I	D3-22-07	OQ826987	Bangladesh	2022
DENV-3	I	D3-22-08	OQ826988	Bangladesh	2022
DENV-3	I	D3-22-09	OQ826989	Bangladesh	2022
DENV-3	I	D3-22-10	OQ826990	Bangladesh	2022
DENV-3	I	D3-22-11	OQ826991	Bangladesh	2022
DENV-3	I	D3-22-12	OQ826992	Bangladesh	2022
DENV-3	I	D3-22-13	OQ826993	Bangladesh	2022
DENV-3	I	D3-22-14	OQ826994	Bangladesh	2022
DENV-3	I	D3-22-15	OQ826995	Bangladesh	2022
DENV-3	I	D3-22-16	OQ826996	Bangladesh	2022
DENV-3	I	D3-22-17	OQ826997	Bangladesh	2022
DENV-3	I	D3-22-18	OQ826998	Bangladesh	2022
DENV-3	I	D3-22-19	OQ826999	Bangladesh	2022
DENV-3	I	D3-22-20	OQ827000	Bangladesh	2022
DENV-3	I	D3-22-21	OQ827001	Bangladesh	2022
DENV-3	I	D3-22-22	OQ827002	Bangladesh	2022

DENV-3	I	D3-22-23	OQ827003	Bangladesh	2022
DENV-3	I	D3-22-24	OQ827004	Bangladesh	2022
DENV-3	I	D3-22-25	OQ827005	Bangladesh	2022
DENV-3	I	D3-22-26	OQ827006	Bangladesh	2022
DENV-3	I	D3-22-27	OQ827007	Bangladesh	2022
DENV-3	I	D3-22-28	OQ827008	Bangladesh	2022
DENV-3	I	D3-22-29	OQ827009	Bangladesh	2022
DENV-3	I	D3-22-30	OQ827010	Bangladesh	2022
DENV-3	I	D3-22-31	OQ827011	Bangladesh	2022
DENV-3	I	D3-22-32	OQ827012	Bangladesh	2022
DENV-3	I	D3-22-33	OQ827013	Bangladesh	2022
DENV-3	I	D3-22-34	OQ827014	Bangladesh	2022
DENV-3	I	D3-22-35	OQ827015	Bangladesh	2022
DENV-3	I	D3-22-36	OQ827016	Bangladesh	2022
DENV-3	I	D3-22-37	OQ827017	Bangladesh	2022
DENV-3	I	D3-22-38	OQ827018	Bangladesh	2022
DENV-3	I	D3-22-39	OQ827019	Bangladesh	2022

Table S4. Age and sex distribution of study population (serotype-positive)

Age (Years)	2018 (n=127)		2019 (n=86)		2020 (n=01)		2021 (n=178)		2022 (n=103)	
	Male (%)	Female (%)	Male (%)	Female (%)	Male (%)	Female (%)	Male (%)	Female (%)	Male (%)	Female (%)
**<1**	10 (7.9)	5 (3.9)	2 (2.3)	2 (2.3)	0	0	1 (0.6)	2 (1.1)	2 (1.94)	1 (0.97)
**1 – 10**	18 (14.2)	12 (9.4)	18(20.9)	15(17.4)	0	0	12 (6.7)	10 (5.6)	18(17.48)	13(12.62)
**11-20**	13 (10.2)	6 (4.7)	11(12.8)	11(12.8)	0	0	33(18.5)	12 (6.7)	12(11.65)	7 (6.8)
**21-30**	6 (4.7)	7 (5.5)	3 (3.5)	4 (4.6)	0	0	24(13.5)	8 (4.5)	10(9.71)	7 (6.8)
**31-40**	12 (9.4)	8 (6.3)	4 (4.6)	3 (3.5)	0	0	24(13.5)	16 (9)	8 (7.77)	8 (7.77)
**41-50**	10 (7.9)	7 (5.5)	3 (3.5)	1 (1.2)	1(100)	0	11 (6.2)	7 (3.9)	6 (5.83)	4 (3.88)
**51-60**	6 (4.7)	2 (1.6)	6 (7)	2 (2.3)	0	0	4 (2.2)	5 (2.8)	0	1 (0.97)
**61-70**	2 (1.6)	1 (0.8)	0	1 (1.2)	0	0	1 (0.6)	3 (1.7)	2 (1.94)	1 (0.97)
**>70**	1 (0.8)	1 (0.8)	0	0	0	0	1 (0.6)	4 (2.2)	2 (1.94)	1 (0.97)
Total	78 (61.42)	49(38.58)	47(54.65)	39(45.35)	1(100)	0	111(62.36)	67(37.64)	60(58.25)	43(41.75)

Table S5. Severity of sequenced serotypes during the period 2018–2022

Year	Sequenced Serotypes and severity distribution						
	Classical			Severe			Total sequenced serotype (%)
	DENV1 (%)	DENV2 (%)	DENV3 (%)	DENV1 (%)	DENV2 (%)	DENV3 (%)	
2018	5 (8.47)	26 (44.07)	23 (38.98)	0	4 (6.78)	1 (1.69)	59 (32.96)
2019	4(9.76)	0	21 (51.22)	0	0	16 (39.02)	41 (22.91)
2020	0	0	0	0	0	0	0
2021	0	0	34 (85)	0	0	6 (15)	40 (22.35)
2022	0	0	35 (89.74)	0	0	4 (10.26)	39 (21.79)
Total	9 (5.03)	26 (14.53)	113 (63.13)	0	4 (2.23)	27 (15.08)	179 (100)
